# Cardiac pathology in spinal muscular atrophy: a systematic review

**DOI:** 10.1186/s13023-017-0613-5

**Published:** 2017-04-11

**Authors:** C. A. Wijngaarde, A. C. Blank, M. Stam, R. I. Wadman, L. H. van den Berg, W. L. van der Pol

**Affiliations:** 1 Department of Neurology and Neurosurgery, F02.230, Rudolf Magnus Institute of Neuroscience, University Medical Center Utrecht, Heidelberglaan 100, 3508 GA Utrecht, The Netherlands; 2 Department of Pediatric Cardiology, Wilhelmina Children’s Hospital, University Medical Center Utrecht, Utrecht, The Netherlands

**Keywords:** Spinal muscular atrophy, SMA, Werdnig-Hoffmann, Kugelberg-Welander, Cardiac pathology, Cardiac abnormalities

## Abstract

**Background:**

Hereditary proximal spinal muscular atrophy (SMA) is a severe neuromuscular disease of childhood caused by homozygous loss of function of the survival motor neuron (SMN) 1 gene. The presence of a second, nearly identical SMN gene (SMN2) in the human genome ensures production of residual levels of the ubiquitously expressed SMN protein. Alpha-motor neurons in the ventral horns of the spinal cord are most vulnerable to reduced SMN concentrations but the development or function of other tissues may also be affected, and cardiovascular abnormalities have frequently been reported both in patients and SMA mouse models.

**Methods:**

We systematically reviewed reported cardiac pathology in relation to SMN deficiency. To investigate the relevance of the possible association in more detail, we used clinical classification systems to characterize structural cardiac defects and arrhythmias.

**Conclusions:**

Seventy-two studies with a total of 264 SMA patients with reported cardiac pathology were identified, along with 14 publications on SMA mouse models with abnormalities of the heart. Structural cardiac pathology, mainly septal defects and abnormalities of the cardiac outflow tract, was reported predominantly in the most severely affected patients (i.e. SMA type 1). Cardiac rhythm disorders were most frequently reported in patients with milder SMA types (e.g. SMA type 3). All included studies lacked control groups and a standardized approach for cardiac evaluation.

The convergence to specific abnormalities of cardiac structure and function may indicate vulnerability of specific cell types or developmental processes relevant for cardiogenesis. Future studies would benefit from a controlled and standardized approach for cardiac evaluation in patients with SMA.

**Electronic supplementary material:**

The online version of this article (doi:10.1186/s13023-017-0613-5) contains supplementary material, which is available to authorized users.

## Background

Hereditary proximal spinal muscular atrophy (SMA) is an important genetic cause of infantile mortality and childhood disability. Degeneration of α-motorneurons in the ventral horns of the spinal cord is the most salient feature but other organs, in particular the heart, may also be affected as suggested by numerous case reports [1, 2].

SMA is caused by deficiency of the survival motor neuron (SMN) protein due to homozygous loss of function of the SMN1 gene. The human SMN locus contains a second, nearly identical, SMN copy (SMN2) that contains a critical point mutation in exon 7, resulting in exclusion of exon 7 from most SMN2 mRNA transcripts. SMN2 therefore produces residual levels of full length SMN2 mRNA and functional SMN protein [3–7]. SMN protein is ubiquitously expressed and is part of multiprotein complexes that probably have both general and motor neuron specific functions, including small nuclear ribonucleic protein (snRNP) assembly, pre-mRNA splicing, post-transcriptional gene regulation, axonal mRNA transport, ubiquitination homeostasis, maintenance and neuronal differentiation of embryonic stem cells and embryonic organ development [6, 8–13]. Variation in SMN2 copy numbers, which partly explains differences in SMN protein levels between patients, is the most important modifier of SMA severity. The severity spectrum encompasses prenatal SMA (type 0), infantile onset severe SMA (type 1), an intermediate form (SMA type 2), childhood onset SMA (type 3), and adult onset SMA (type 4). Higher copy numbers are associated with milder forms of SMA [5, 6].

The identification of non-neuromuscular complications of severe SMA, including disorders of the heart and cardiovascular system, may help to elucidate pathogenic pathways and are furthermore of increasing clinical importance since therapies that aim to attenuate or reverse SMN deficiency may be introduced soon.

To study the evidence for an association of SMA with cardiac pathology in more detail, we performed a systematic review of the available clinical and experimental literature.

## Methods

### Search

We searched MEDLINE and Embase for articles on SMA and cardiac pathology published up to January 31st 2016, using a combination of the following terms: ‘spinal muscular atrophy’, ‘Werdnig Hoffmann’ and ‘Kugelberg Welander’, ‘heart’, ‘cardiac’, and ‘ECG’. Numerous word variations were included and specific types of cardiac abnormalities were added to the search, including ‘congenital heart disease’, ‘atrial septal defect’, ‘ventricular septal defect’, ‘cardiac malformations’, and ‘arrhythmias’, in order to identify as many relevant articles as possible. The query that retrieved the largest number of relevant results was used and is shown in Table 1.

**Table 1:** Details on systematic search

Search terms used for PubMed search	
Topic	Query^a^
SMA	(“spinal muscular atrophy” OR muscular atroph^b^ OR “werdnig hoffmann” OR “kugelberg welander”)
Cardiac Pathology	AND (“heart” OR “cardiac” OR cardiol^b^ OR ventric^b^ OR “septum” OR “ECG”)

^a^Similar terms were used in all searches, tailored to the specific requirements of each search engine. The addition of specific cardiac abnormalities did not retrieve relevant additional results and were therefore excluded from our final query

^b^ *indicates that word variations of the search term were also searched*

The MEDLINE database was searched using PubMed. In addition, Scopus, OvidSP, and Web of Science were used to obtain as many relevant original papers as possible. For OvidSP, the following resources were selected: ‘MEDLINE’, ‘OLD MEDLINE’ and ‘MEDLINE In-Process’. Similar terms were used for all searches, tailored to the specific requirements of each search engine. No field limitations or language restrictions were applied. We used indexed search terms, if applicable, to ensure inclusion of relevant related terms. MeSH-indexed terms were not used in order to prevent missing recently published articles that had not yet been MeSH-indexed at the time of our search. For articles possibly relevant to our search but unavailable online, we searched university library catalogues using ‘Picarta’ (http://www.picarta.org/) to check for offline availability, and contacted the author(s) of the original publications if e-mail or correspondence addresses were available.

### Selection of relevant articles

**Fig. 1:**
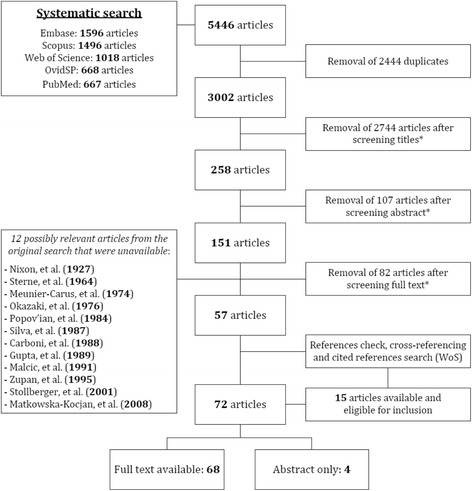
Flowchart of search and selection process. Summary of search and selection process of eligible articles for inclusion. *: predefined inclusion and exclusion criteria were applied, as shown in Table 2. WoS: ‘Web of Science’

Two of the authors [CAW, MS] independently conducted the search and selection processes. After screening title and abstract of all obtained articles, potentially relevant papers were screened full text using predefined inclusion and exclusion criteria (Table 2). Both clinical and experimental studies of patients and mouse models were included. We also systematically checked the references of all included papers and used Thomson Reuters’ ‘Web of Science’ for a cited references search and a related articles search, to ensure identification of all relevant literature. Details of the search and selection process are summarized in Fig. 1.

**Table 2:** Criteria used for critical selection of papers retrieved from our search

Applied criteria for the selection of relevant papers	
Inclusion criteria	Diagnosis of SMA types 1–4^a^ or SMA mouse model; presence of cardiac abnormalities; original study with identifiable case(s).
Exclusion criteria	No diagnosis of SMA or substantial doubts about diagnosis; diagnosis of non 5q-SMA (e.g.: SMARD, distal SMA); no cardiac pathology present; SMA with additional chromosomal abnormalities associated with (congenital) heart disease (e.g. trisomy 21); cardiac abnormalities due to medication or in moribund patients (e.g. bradycardia); redundant publication of previously reported case(s); congress reports; mouse model of non 5q-SMA (e.g. IGHMBP2 model); animal research other than mouse models; only abstract available with unidentifiable cases.

^a^This also includes the SMA subtypes, e.g. ‘type 0’, ‘type 1a’, ‘type 1b’, ‘type 3a’ and ‘type 3b’. *SMA* spinal muscular atrophy; *SMARD* spinal muscular atrophy with respiratory distress; *IGHMBP2* Immunoglobulin Mu Binding Protein 2

### Data extraction

After all relevant data was extracted from the selected papers [by CAW], two authors [CAW and ACB] independently categorized structural (congenital) cardiac defects using the 2012 version of the European Paediatric Cardiac Code (International Paediatric and Congenital Cardiac Code (IPCCC) Short List) [14, 15] that distinguishes 8 groups, based on affected anatomical areas of the heart (Table 3). We also classified abnormalities of cardiac rhythm using the system suggested by Korpas [16], which is based upon mechanisms of origin, i.e.: arrhythmias due to abnormal impulse initiation or abnormal impulse conduction. Impulse initiation disorders were further subdivided into 3 groups, based upon the area of the cardiac conduction system involved: sinoatrial (sinus) node, supraventricular, or ventricular (Table 5). Initial classification disagreements were resolved by consensus. A comprehensive overview of all retrieved cases of SMA patients with cardiac pathology is shown in Additional file 1: Tables S1–S3.

**Table 3:** Classification of structural cardiac defects in SMA type 1 patients

Diagnostic groups		Reported occurrence
1.	Abnormalities of position and connection of heart	2x
2.	Tetralogy of Fallot and variants	1x
3.	Abnormalities of great veins	–
4.	Abnormalities of atria and atrial septum	23x
5.	Abnormalities of atrioventricular valves and atrioventricular septal defect	3x
6.	Abnormalities of ventricles and ventricular septum	17x
7.	Abnormalities of ventriculo-arterial valves and great arteries	7x
8.	Abnormalities of coronary arteries, arterial duct and pericardium	6x

Reported structural (congenital) cardiac abnormalities were classified into 8 groups, in accordance with the European Paediatric Cardiac Code (IPCCC short list) [14, 15]. Table 3 shows details on a total of 42 patients, some of whom had more than one structural cardiac abnormality

The small number of both patients and SMA model mice with histological abnormalities of cardiac tissue precluded the use of available classification systems, nor was it possible to classify cardiac abnormalities in SMA mouse models due to significant methodological differences between studies. A comprehensive overview of all included SMA mouse models is shown in Additional file 1: Table S5.

## Results

We retrieved 3002 articles with our initial search. After selection, 72 articles met our predefined inclusion criteria, including 4 articles of which only the abstract was available [17–20]. These abstracts contained sufficient detailed information and were included for further analysis. We were unable to obtain full text or detailed abstracts of 15 possibly relevant articles. Twelve of these articles were identified in the original search, whilst the other 3 were found through the related articles search (Additional file 1: Table S4, [21–35]).

We identified a total of 264 published cases of SMA patients with cardiac pathology. Seven studies contained descriptions of patients with several SMA types, 28 studies of SMA type 1 only and 23 studies of SMA type 3 only. We found a total of 14 studies on cardiac pathology in SMA mouse models (Additional file 1: Tables S1–S3 and S5).

### Cardiac pathology in patients with SMA type 1

We identified 77 patients with SMA type 1 (‘Werdnig-Hoffmann Disease’) and cardiac pathology [36–69]. Most studies used well defined clinical criteria for the diagnosis of SMA. Tests for homozygous SMN1 deletion were performed in 36 (47%) patients and confirmed in 31: five patients did not have a homozygous SMN1 deletion. It was not specified whether these patients had intragenic SMN1 point mutations, or a different neuromuscular disorder. The diagnosis in these 5 patients was based upon clinical characteristics combined with supportive information from muscle biopsies (*n* = 5), EMG (*n* = 4) and autopsy findings (*n* = 2) [44, 50, 54, 59].

Thirty-three out of 77 (43%) patients had electrocardiogram (ECG) abnormalities. ECGs of 15 patients (19%) showed severe symptomatic bradycardias, defined as a heart rate of less than 40 beats per minute. Baseline tremors were reported in the other 18 (23%) patients. These ECG baseline tremors were initially interpreted as abnormalities or even as being suggestive of cardiac pathology, but nowadays they are interpreted as artefacts due to peripheral muscle tremors. Two patients were diagnosed with a ‘late form of Werdnig-Hoffmann’ but it is unclear whether these two patients had SMA type 2 or 3 and were misclassified or had a relatively mild form of SMA type 1 (i.e. type 1c) with longer survival [70, 71]. The two children, aged 10 and 12, had echocardiographic abnormalities compatible with left ventricular hypertrophy (Additional file 1: Table S1, patients 8 and 9) [39].

The remaining 42 patients (55%) with SMA type 1 had structural cardiac defects. We used the IPCCC diagnostic classification system as outlined in Table 3 [14, 15].

**Fig. 2:**
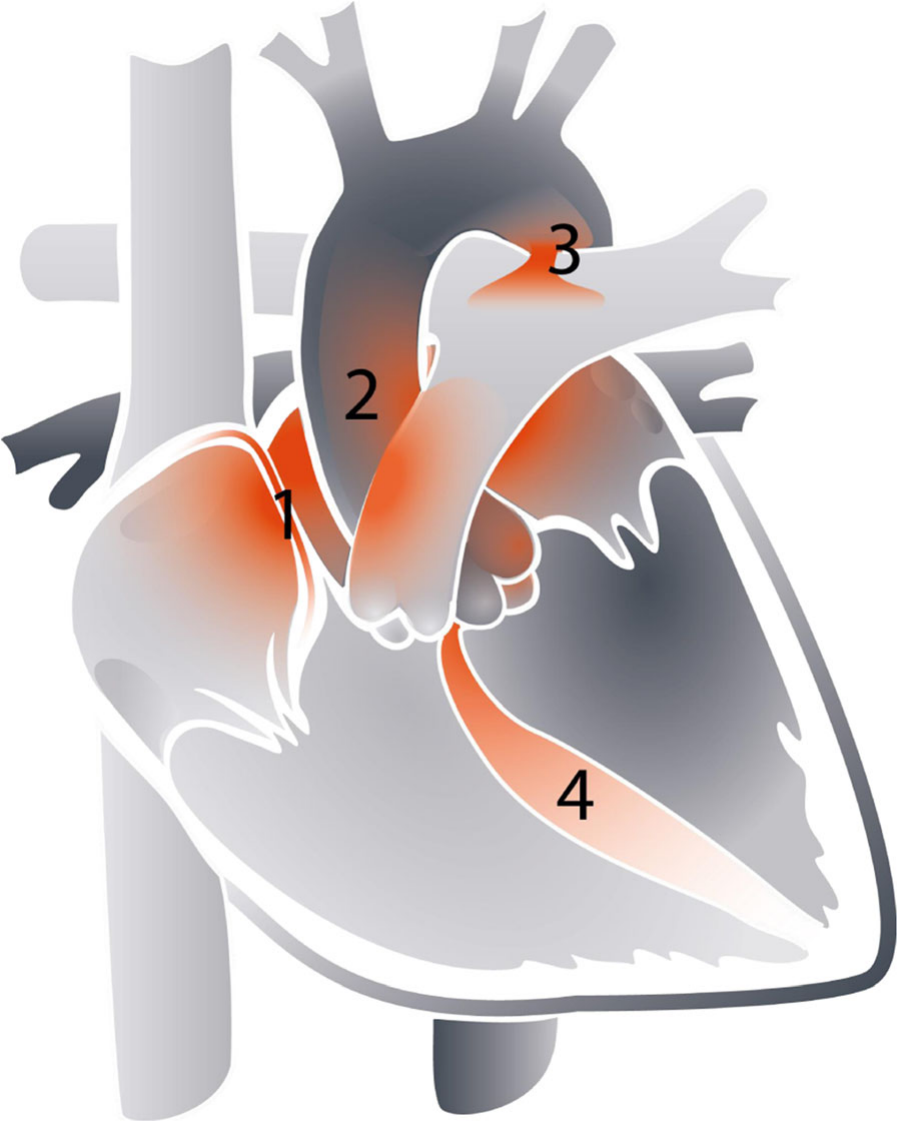
Structural cardiac pathology in SMA type 1. Areas of the heart frequently reported to show cardiac defects in patients with SMA type 1 (*n* = 42) are shown in color. Rarely affected and unaffected areas are shown in shades of grey. Numbers indicate 1: atrial septum; 2: cardiac outflow tract; 3: patent ductus arteriosus; 4: ventricular septum

Twenty-one of the 42 patients (50%) had a single structural abnormality of the heart. This included 11 (26%) patients with an atrial septal defect (ASD) and 5 (12%) with an isolated ventricular septal defect (VSD). The other 21 patients (50%) had multiple structural cardiac abnormalities. The combination of an ASD (IPCCC group 4) and VSD (IPCCC group 6) was reported relatively frequently (*n* = 5; 12%). Hypoplastic left heart syndrome was also found in 5 (12%) patients. Additional file 1: Table S1 includes details on all included patients. Figure 2 illustrates the reported structural cardiac pathology in SMA type 1.

We next assessed the relationship between SMN2 copy numbers and the severity of cardiac pathology. SMN2 copy numbers were available for only 13 of the 77 patients with SMA type 1 (17%). Ten patients had 1 SMN2 copy and 3 had 2 SMN2 copies (Table 4) [46, 53, 56, 63, 65–68].

**Table 4:** SMN2 copy numbers in patients with SMA type 1 and cardiac abnormalities

Reference	Patient no.	SMN2 copy number	Cardiac pathology	IPCCC diagnostic group(s)
Devriendt (1996) [46]	1	1 copy	Small VSD	Group 6
Macleod (1999) [53]	2	1 copy ^χ^	ASD	Group 4
	3	1 copy ^χ^	ASD and mitral hypoplasia	Groups 4 and 5
García-Cabezas (2004) [56]	4	1 copy	ASD (secundum type)	Group 4
Rüdnik-Schöneborn (2008) [63]	5	1 copy	Large ASD (sinus venosus type), multiple VSDs and PDA	Groups 4, 6 and 8
	6	1 copy	Common atrium (i.e. a very large ASD) and PDA	Groups 4 and 8
	7	1 copy	Large ASD (secundum type) and VSD	Groups 4 and 6
	8	2 copies	Small VSD and PDA	Groups 6 and 8
Lumaka (2009) [65]	9	2 copies	ASD (secundum type)	Group 4
Rüdnik-Schöneborn (2010) [66]	10	1 copy	Large ASD, mild pulmonary and mild aortic stenosis	Groups 4 and 7
	11	1 copy	AVSD	Group 5
Parra (2012) [67]	12	1 copy	HLHS	Group 6
Ekici (2012) [68]	13	2 copies	Dextrocardia and Tetralogy of Fallot	Groups 1 and 2

The number of SMN2 copies of 13 patients with SMA type 1

*Abbreviations*: *IPCCC* International Paediatric and Congenital Cardiac Code; *VSD* ventricular septal defect; *ASD* atrial septal defect; *PDA* patent ductus arteriosus; *AVSD* atrioventricular septal defect; *HLHS* hypoplastic left heart syndrome. χ = SMN2 copy numbers were calculated based on the SMN:MPZ ratio provided by the authors in the original publication [53]

### Cardiac pathology in patients with SMA type 2

We found 5 studies with a total of 63 patients with SMA type 2 and cardiac pathology [37, 40, 41, 49, 72]. No genetic tests to confirm the diagnosis were performed. Clinical characteristics, sometimes combined with documented muscle biopsy (*n* = 21) and EMG results (*n* = 8), were used for diagnosis. Many of the original publications mentioned the use of ancillary investigations to support the diagnosis (e.g. EMG, muscle biopsy, autopsy) but did not further specify the tests.

All 63 patients had ECG baseline tremors (Additional file 1: Table S2). A small number of patients also had one or more additional ECG abnormalities [72]. This included disorders of impulse initiation (*n* = 2; both with a sinus tachycardia), disorders of impulse conduction (*n* = 2; both with a right bundle branch block), signs of atrial enlargement (*n* = 3) or ventricular enlargement (*n* = 4), signs of myocardial damage (abnormal Q-waves *n* = 2, ST-changes *n* = 3), or signs of ventricular hypertrophy (*n* = 7).

### Cardiac pathology in patients with SMA type 3

We identified 124 patients with SMA type 3 (‘Kugelberg-Welander Disease’, ‘Wolfhart-Kugelberg-Welander Disease’) and cardiac pathology [17–20, 36, 37, 40, 41, 47, 49, 72–91]. Genetic tests to confirm homozygous deletion of SMN1 were performed in 5 patients (4%) and confirmed in 4 [47, 86, 88, 89, 91]. It remains unclear whether this one patient might have had a hemizygous deletion in combination with an intragenic SMN1 point mutation, or ‘non-5q’ SMA [88]. SMN2 copy numbers were not available for any of the patients (Additional file 1: Table S3).

Sixty-seven (54%) of the 124 patients had tremors of the ECG baseline only. Six patients (5%) had both ECG abnormalities and structural cardiac pathology [80, 82]. One presented with mitral and tricuspid valve prolapse (IPCCC group 5), five patients had a prolonged QT time interval combined with one or more structural abnormalities: mitral valve prolapse (*n* = 3, IPCCC group 5), a hypertrophic interventricular septum (*n* = 2, IPCCC group 6) or a hypertrophic (*n* = 1) or atrophic (*n* = 1) posterior ventricular wall. One of these patients also had a diminished left ventricular end diastolic volume.

Two patients had structural abnormalities of the heart without abnormalities of the cardiac rhythm. One, with genetically confirmed SMA type 3a, had a complex cardiac malformation consisting of an ASD, L-transposition of the great arteries, functional single ventricle and a patent ductus arteriosus (IPCCC groups 2, 4, 6, and 8) [47]. The other patient had an ASD (ostium secundum type, IPCCC group 2) [90].

Thirty-nine (31%) of the 124 patients with SMA type 3 had abnormalities of the cardiac rhythm only. With details provided in the original publications we were able to further classify cardiac rhythm abnormalities of 24 patients (19%), using a classification system suggested previously (Table 5) [16].

**Table 5:** Arrhythmias in SMA type 3

Arrhythmias in patients with SMA type 3		
*Arrhythmia type*	*Anatomical site of origin*	*Reported ECG abnormalities* *(number of times observed in patients with SMA type 3)*
Impulse initiation disorder	Sinus/SA node initiation disorders	Sinus arrest (2)
		Sinus dysfunction (1)
		Sinus tachycardia (1)
	Supraventricular initiation disorders	Atrial fibrillation (5)
		AV junctional rhythm (5)
		Supraventricular extrasystoles (2)
		Atrial flutter (2)
		Atrial tachycardia (1)
	Ventricular initiation disorders	Ventricular extrasystoles (2)
		Non-sustained ventricular tachycardias (2)
Impulse conduction disorders	*n/a*	Left anterior hemiblock (4)
		AV-block (n.o.s.) (3)
		Right bundle branch block (3)
		1st degree AV block (3)
		Complete AV block (2)
		2nd degree AV block, Mobitz I (Wenckebach) (1)
		Prolonged junctional recovery time (1)

Reported arrhythmias in patients with SMA type 3 (*n* = 24). Some patients had more than one ECG abnormality. Also see Additional file 1: Table S3

*Abbreviations*: *SA* sinoatrial; *AV* atrioventricular; *n.o.s*. not otherwise specified; *n*/*a* not applicable

Additionally, 9 patients (7%) were reported with ECG signs suggestive of myocardial damage (e.g. Q-waves or ST-changes) [72, 73, 76, 77, 80, 81], two patients had systolic pump function abnormalities [86, 88], and 2 others had ECG abnormalities that could not be classified further [20, 83].

Finally, information on histological cardiac abnormalities from autopsy (*n* = 5) or cardiac biopsy (*n* = 3) was available for 8 patients (6%). Myocardial fibrosis was the most frequent finding (*n* = 4) [19, 73, 75–78, 89]. Two studies contained detailed descriptions of the myocardial histology, including findings of deranged, atrophic and degenerated myocytes and myocardial fibers [75, 78]. Ultrastructural myocardial changes, such as focal degeneration of myocardial cells, were also noted [75].

### Cardiac pathology in SMA mouse models

We found 14 studies that reported the presence of cardiac pathology in SMA mouse models (Additional file 1: Table S5) [10, 92–104]. Methods used to evaluate and classify cardiac involvement differed considerably between studies, complicating a comparison of reported outcomes. The most important macroscopic findings were decreased heart size including decreased left ventricular (LV) mass [10, 92–94, 96, 102, 104]. This finding could at least partially be explained by reduced body size and weight. Furthermore, reduced thickness of the LV wall and interventricular septum (IVS) were frequently reported [10, 94, 96, 99, 100, 102]. Abnormal cardiogenesis of the IVS, LV and arterial walls, which also accounts for thinning and partial flattening of the cardiac arterial walls, was suggested as a possible underlying cause in one study [94].

The most prominent microscopic finding was myocardial fibrosis [94, 96, 98, 100]. Other abnormalities included vascular remodelling, including decreased numbers of coronary capillaries, and ultrastructural changes, e.g. abnormal expression of postnatal cardiac development markers indicating loss of contractile components (Additional file 1: Table S5).

Microscopic abnormalities of the cardiac autonomic nervous system (ANS) were also reported, including reduced neuronal branching and presence of thinner cardiac sympathetic ANS nerves [93, 95]. Abnormalities of the cardiac ANS or cardiac rhythm were reported in most studies. Bradyarrhythmias were reported in almost all studies [92–97, 101, 103, 104]. Few studies included detailed information on murine cardiac rhythm, which precludes further classification. The available data suggest that both disorders of impulse initiation and impulse conduction can be found in mouse models of SMA.

The main finding indicating reduced cardiac function was a significant reduction in pumping efficiency, i.e. reduced stroke volume and cardiac output, mainly due to left ventricular dysfunction [92, 93, 104]. All findings are summarized in Additional file 1: Table S5.

## Discussion

Vulnerability to SMN deficiency may not be confined to motor neurons. Cardiovascular abnormalities are among the most frequently reported non-neuromuscular complications in SMA [2]. In this systematic review, we identified 264 published possible cases of SMA with cardiac abnormalities and 14 studies reporting cardiac involvement in SMA mouse models. Structural cardiac pathology was almost exclusively reported in patients with SMA type 1, while acquired cardiac pathology, including arrhythmias and conduction abnormalities, were reported more frequently in less severely affected patients. Detailed classification of the reported abnormalities suggests convergence to specific pathologies in patients with SMA that may be linked to downstream effects of SMN deficiency. We did not identify large controlled studies that indicate the presence of cardiac pathology in SMA, preventing a definite conclusion as to whether the incidence of cardiac abnormalities is increased in SMA.

Structural cardiac abnormalities in SMA type 1 were almost exclusively defects of atrial and ventricular septa and/or defects of the cardiac outflow tract. Ventricular septal defects (VSD), pulmonary stenosis, a patent ductus arteriosus (PDA), and atrial septal defects (ASD) are, however, the most common structural cardiac abnormalities in newborns, with a reported incidence of approximately 1% [105–110]. Low SMN protein levels may increase the odds of abnormal cardiac development. This hypothesis is supported by several observations: interventricular septum abnormalities were also observed in animal models of severe SMA, and abnormal embryonic cardiogenesis induced by low SMN protein levels was identified as a possible underlying cause in one study [94]. Moreover, there was an over-representation of patients with SMA type 1 and cardiac defects who had only one SMN2 copy, which is associated with the lowest residual SMN protein levels that are compatible with life at birth [5]. The association between the lowest SMN2 copy number and occurrence of non-neuromuscular pathology, including cardiac abnormalities, has been suggested previously [63].

Disturbances of cardiac rhythm were a second abnormality reported across the spectrum of SMA severity, i.e. in SMA types 1–3. Leaving out baseline tremors, which are to be considered an artefact caused by the characteristic peripheral tremor in patients with SMA, impulse initiation disorders were the most common cardiac rhythm abnormalities. Taking into account the very low reported incidence of for example atrial flutter or atrial fibrillation in patients under the age of 50 years [111, 112], impulse initiation disorders occurred at a strikingly young age in patients with SMA (atrial flutter, *n* = 2, ages 24 and 49 years [77, 80]; atrial fibrillation, *n* = 4, reported ages ranging from 29 to 35 years [17, 19, 88]). This may suggest a developmental origin associated with SMN deficiency. In theory, both dysfunction of either the cardiac electrical conduction system or the ANS, which influences cardiac rhythm in vivo, may underlie cardiac arrhythmias [113]. Significant abnormalities of the cardiac ANS were also found in SMA mouse models [93, 95].

Myocardial fibrosis was reported in 8 patients and may contribute to arrhythmias in SMA [19, 73, 76, 89]. Fibrosis of the myocardium was also a frequent finding in both severe and intermediate SMA mouse models, in which arrhythmias were virtually omnipresent. Bradycardia was reported most often, due to delays in the cardiac electrical conduction system, causing various types of atrioventricular and bundle branch blocks. It should be noted that myocardial fibrosis is a hallmark of normal aging [114], and the limited number of patients precludes a definite conclusion whether impulse conduction disorders in SMA are caused by presenile cardiac fibrosis secondary to SMN deficiency.

There are several other possible explanations how SMN deficiency causes cardiac abnormalities, including specific mRNA-splicing defects that could interfere with normal cardiac development [11, 115]. Low SMN levels have already been shown to influence embryonic organ development in animal models, including cardiogenesis [2, 6, 116]. Furthermore, very low levels of SMN protein may predispose to dysfunction of specific cell types other than alpha-motor neurons, that are involved in cardiogenesis [117–119]. A potential candidate cell type is the neural crest cell (NCC), as a subset of NCCs migrate and differentiate into cardiac neural crest cells (cNCCs) that are involved in development of the musculoconnective tissue (tunica media) of the great vessels, cardiac outflow tract septa (dividing the conotruncus into the aorta and pulmonary trunk) and, to some extent, septation of the atria and ventricles [120–126]. SMN protein deficiency may alter the function of downstream signalling pathways that are important for the migratory process of NCCs [123]. Furthermore, although the cardiac electrical conduction system itself originates from cardiomyocytes [127], the cardiac ANS that contributes to arrhythmias, develops from NCCs [113].

Several limitations of this systematic review need to be addressed. First, we cannot exclude the possibility of publication bias towards cases with particular findings or with severe forms of SMA and heart disease. Published cases may, therefore, not be representative of all cardiac pathology in SMA and single cases could have been missed if they were not represented in the databases used. However, given the relatively large number of patients included, it is unlikely that these cases would have substantially influenced our overall findings. Furthermore, publications and reports differed significantly in clinical detail and the time of diagnosis. Many studies were published before genetic testing for homozygous SMN1 deletion became widely available (i.e.: cases before 1995) which leaves open the possibility of inclusion of disorders other than SMA, in particular for SMA types 2 and 3. The cases of patients with SMA and heart disease included in our work were published between the late 1960s [73] and 2015 [91]. During this period, significant modifications of diagnostic criteria and classifications of SMA types occurred [128, 129]. Although these changes are largely irrelevant with regard to observing a cardiac abnormality in a patient with SMA, we had to assume the correct diagnosis of SMA (in the absence of genetic confirmation of the diagnosis) and severity in some patients. With a view to addressing these issues, at least in part, we reviewed all available clinical data of included cases (Additional file 1: Tables S1–S3) in an attempt to maximise diagnostic accuracy. Finally, considerable differences in the diagnostic methodology for cardiac evaluation, ranging from a limited number of diagnostic tools to assess cardiac pathology, to a more comprehensive combination of ECG, radiographs, echocardiography, or autopsy, clearly results in differences in quality of observations between studies.

## Conclusions

On the basis of the data available, if present, structural abnormalities of the heart are predominantly expected in the more severely affected SMA patients (i.e. SMA type 1), disturbances of the cardiac rhythm in the more mildly affected patients (i.e. SMA types 2 and 3). Future studies would benefit greatly from a controlled, standardized, uniform, and comprehensive protocol for cardiac work-up of genetically confirmed cases of SMA.

## Additional file

Additional file 1:
**Tables S1**, **S2**, and **S3**: provide an overview of all included studies and all individual cases of patients with SMA and cardiac pathology. **Table S4:** overview of articles (*n* = 12) possibly relevant to our systematic search of which no full text or detailed abstract was available. **Table S5.** Overview of SMA mouse models with cardiac pathology. (DOCX 153 kb)

## Abbreviations

ANS: Autonomic nervous system
ASD: Atrial septal defect
AV: Atrioventricular
AVSD: Atrioventricular septal defect
cNCC: Cardiac neural crest cell
ECG: Electrocardiogram
EMG: Electromyogram
HLHS: Hypoplastic left heart syndrome
IGHMBP2: Immunoglobulin Mu Binding Protein 2
IPCCC: International Pediatric Cardiac Code
IVS: Intraventricular septum
LV: Left ventricular
mRNA: Messenger-ribonucleic acid
NCC: Neural crest cell
PDA: Patent ductus arteriosus
SA: Sinoatrial
SMA: Spinal muscular atrophy
SMARD: Spinal muscular atrophy with respiratory distress
SMN: Survival motor neuron
snRNP: Small nuclear ribonucleic protein
VSD: Ventricular septal defect
