# Supplementary material for: Cardiac pathology in spinal muscular atrophy: a systematic review
Source: Orphanet J Rare Dis. 2017 Apr 11;12:67. doi: 10.1186/s13023-017-0613-5 (PMC5387385; doi:10.1186/s13023-017-0613-5)
Supplement: Additional file 1: — Tables S1, S2, and S3: provide an overview of all included studies and all individual cases of patients with SMA and cardiac pathology. Table S4: overview of articles (n = 12) possibly relevant to our systematic search of which no full text or detailed abstract was available. Table S5. Overview of SMA mouse models with cardiac pathology. (DOCX 153 kb) [file 13023_2017_613_MOESM1_ESM.docx]

**Additional file**

**Table S1: overview of included studies on cardiac pathology in SMA type 1 (Werdnig-Hoffmann disease).**

| **Reference** | **Case no.** | **Patient characteristics** | | | **SMN1 gene status** | **SMN2 copy no.** | **Cardiac pathology** |
| --- | --- | --- | --- | --- | --- | --- | --- |
|  |  | **Sex (m/f)** | **Age (years)** | **SMA type^♦^** |  |  |  |
| Russman (**1979**)[21] | 1–3 | n/a | n/a  *‘floppy at birth’* | Type 1 | n/a | n/a | All 3 had ECG baseline tremors |
| Dawood (**1983**)[22] | 4–5 | n/a | Onset from birth.  *Mean age at presentation: 5 months (range  1–15 months)* | Type 1 | n/a | n/a | ECG baseline tremors, more marked in the limb than chest leads.  *Authors mention that only 2 of 10 patients with SMA type 1 showed the ECG baseline tremors.* |
| Towfighi (**1985**)[23] | 6  (*patient 1*) | M | 0 months  *Prenatal onset, AAD: 9 days.* | Type 1 | n/a | n/a | Large ASD and coarctation of the aorta.  *Specific ASD type not specified by authors.* |
|  | 7  (*patient 2*) | M | 0 months  *Prenatal onset, AAD: 3 months.* | Type 1 | n/a | n/a | Large ASD and hypertrophic right ventricle.  *Specific ASD type not specified by authors. The hypertrophic right ventricle could be acquired as well as a primary cardiac abnormality. The details provided in the article are insufficient to further classify the abnormality.* |
| Lintermans (**1987**)[24] | 8* | n/a | 10 | ‘late form of WH’ | n/a | n/a | Hypertrophic left ventricle.  *This could be an acquired or a primary cardiac abnormality. The information provided in the article is insufficient to further classify the reported abnormality.* |
|  | 9* | n/a | 12 | ‘late form of WH’ | n/a | n/a | Hypertrophic left ventricle.  *This could be an acquired or a primary cardiac abnormality. The information provided in the article is insufficient to further classify the reported abnormality.* |
| Coletta (**1989**)[25] | 10  (‘*F.S.’*) | F | 6 months | Type 1 | n/a | n/a | ECG baseline tremors in both limb and precordial (chest) leads. |
|  | 11  (‘*G.A.’*) | M | 7 months | Type 1 | n/a | n/a | ECG baseline tremors in both limb and precordial (chest) leads. |
|  | 12  (‘*Z.C*’) | M | 3 months | Type 1 | n/a | n/a | ECG baseline tremors in limb leads, but not in precordial (chest) leads. |
| Moosa (**1990**)[26] | 13–16 | n/a  *M:F=10:5 for entire group of 15 patients* | n/a  *Onset at birth or within the first 4 months of life.* | Infantile form (Werdnig-Hoffmann) | n/a | n/a | ECG baseline tremors. |
| Moller (**1990**)[27] | 17* | M | 0 months  *Prenatal onset, AAD: 30 minutes after delivery.* | Type 1 | n/a | n/a | Large ASD  *Specific ASD type not specified by authors.* |
|  | 18* | F | 0 months  *Prenatal onset, AAD: 7 weeks.* | Type 1 | n/a | n/a | Large ASD, aortic valve stenosis, hypertrophic right ventricle  *Specific ASD type not specified by authors. The hypertrophic right ventricle could be acquired as well as a primary cardiac abnormality. The details provided in the article are insufficient to further classify the abnormality.* |
|  | 19* | F | 0 months  *Prenatal onset, AAD immediately after delivery.* | Type 1 | n/a | n/a | Large ASD  *Specific ASD type not specified by authors.* |
| Kirkinen (**1994**)[28] | 20 | M | 0 months  *Prenatal onset, AAD: 24 days.* | Type 1 | n/a | n/a | ASD (secundum type) |
| Bürglen (**1995**)[29] | 21  (*patient 1*) | M:F = 2:4 | n/a | Type 1 | no homozygous deletion of SMN1 | n/a | ASD  *Specific ASD type not specified by authors.* |
|  | 22  (*patient 2*) |  | n/a | Type 1 | homozygous del. SMN1 ex 7 | n/a | ASD and VSD  *Specific ASD type not specified by authors.* |
|  | 23  (*patient 3*) |  | n/a | Type 1 | homozygous del. SMN1 ex 7 | n/a | Tricuspid valve atresia with univentricular heart *The univentricular heart is secondary to the trisuspid valve atresia* |
|  | 24  (*patient 4*) |  | n/a | Type 1 | no homozygous deletion of SMN1 | n/a | ASD and aortic coarctation  *Specific ASD type not specified by authors.* |
|  | 25  (*patient 5*) |  | n/a | Type 1 | homozygous del. SMN1 ex 7 | n/a | Partial atrioventricular canal |
|  | 26  (*patient 6*) |  | n/a | Type 1 | homozygous del. SMN1 ex 7 | n/a | Aortic coarctation |
| Rüdnik-Schöneborn (**1996**)[30] | 27  (*patient 21*) | n/a | 0 months  *Ventilated from birth* | Type 1 | ‘SMN gene deletion’ Also deletion of Ag1-CA and C212 markers. | n/a | VSD |
|  | 28  (*patient 25*) | n/a | n/a | Type 1 | ‘SMN gene deletion’ | n/a | VSD |
|  | 29  (*patient 26*) | n/a | n/a | Type 1 | ‘SMN gene deletion’ | n/a | VSD |
| Devriendt (**1996**)[31] | 30 | M | 0 months  *Prenatal onset, AAD: 25 days.* | Type 1 | homozygous del. of SMN1 ex 7 | 1 copy | Small VSD |
| Mulleners (**1996**)[32] | 31  (*case 1*) | F | 0 months  *Onset from birth, AAD: 2 weeks.* | Type 1 | homozygous del. of SMN1 ex 7 | n/a | Large ASD  *Specific ASD type not specified by authors.* |
| Bürglen (**1996**)[33] | 32  (*patient 2*) | F | 0 months  *Prenatal onset, AAD: 6 days.* | ‘Type 0’ | homozygous del. of SMN1 exons 7 + 8 | n/a | Aortic coarctation |
| Huang (**1996**)[34] | 33–38 | M:F = 6:1 | Mean: 6.7 ± 7.5 months | Type 1 | n/a | n/a | ECG baseline tremors in the limb leads. For 1 patient also baseline tremors in precordial (chest) leads. |
| Bingham (**1997**)[35] | 39  (*case 3*) | F | 0 months  *Onset from birth, AAD: 1 month* | Type 1 | no deletion of SMN1 | n/a | ASD  *Specific ASD type not specified by authors.* |
| Rijhsinghani (**1997**)[36] | 40  (*patient 1)* | n/a | *Onset from birth, AAD: 61 days* | Type 1 | ‘positive genetic work-up for SMA type 1’ | n/a | PDA |
| Jong (**1998**)[37] | 41 | M | 3 days at first presentation. *Intubated at 3.5 months* | Type 1 | homozygous SMN1 deletion | n/a | ASD (secundum type) and VSD |
| MacLeod (**1999**)[38] | 42  (*patient 1*) | M | 0 months  *Prenatal onset, AAD: 4 days.* | Type 1 | homozygous del. of SMN1 exon 7 + 8 | 1 copy**^χ^** | ASD  *Specific ASD type not specified by authors.* |
|  | 43  *(patient 3)* | M | 0 months  *Prenatal onset, AAD: 17 days.* | Type 1 | homozygous del. of SMN1 exon 7 + 8 | 1 copy**^χ^** | ASD and mitral hypoplasia  *Specific ASD type not specified by authors.* |
| Courtens (**2002**)[39] | 44 | F | 0 months  *Onset from birth, AAD: 34 days.* | Type 1 | no homozygous deletion of SMN1 | n/a | VSD and a PDA.  *Also secondary (acquired) pulmonary hypertension present.* |
| El-Matary (**2004**)[40] | 45 | M | 0 months  *Prenatal onset, AAD: after withdrawal of ventilator support* | Type 1 | ‘SMA type 1 confirmed with molecular genetic studies’ | n/a | ASD/common atrium, multiple apical VSDs, hypoplastic aortic arch and severe aortic coarctation. Secondary pulmonary hypertension. |
| Garcia-Cabezas (**2004**)[41] | 46 | M | 0 months  *Prenatal onset, AAD: 16 days.* | Type 1 | homozygous deletion of SMN1 | 1 copy | ASD (secundum type) |
| Cook (**2006**)[42] | 47 | M | Age at onset not stated.  *AAD: 8 months* | Type 1 | homozygous del. of SMN1 exon 7 + 8 | n/a | HLHS |
| Menke (**2006**)[43] | 48 | F | 0 months  *Prenatal onset, AAD: 4 days.* | Type 1 | homozygous del. of SMN1 exons 7 + 8 | n/a | HLHS |
| Sarnat (**2007**)[44] | 49^¥^ | M | *Prenatal onset, pregnancy terminated at 20 weeks* | Type 1  *Authors suggest ‘SMA type 0’* | no homozygous deletion of SMN1 | n/a | HLHS |
| Vaidla (**2007**)[45] | 50 | F | 0 months  *Prenatal onset, AAD: 20 days.* | Type 1 | homozygous del. of SMN1 exons 7 + 8 | n/a | ASD and a PDA |
| Bach (**2007**)[46] | 51–65 | n/a | n/a | Type 1 | n/a | n/a | Severe symptomatic bradycardias in 15 patients, defined as less than 40 beats/minute and causing loss of consciousness.  *Authors mention the apparent involvement of the autonomous nervous system.* |
| Menke (2008)[47] | 66^¥,ǂ^ | n/a | *Prenatal onset, pregnancy terminated* | Type 1 | homozygous del. of SMN1 exons 7 + 8 | n/a | HLHS |
| Rüdnik-Schöneborn (**2008**)[48] | 67  (*patient 1*) | M | 0 months  *Prenatal onset, AAD: 9 days.* | Type 1 | homozygous del. of SMN1 | 1 copy | Large ASD (sinus venosus type), multiple VSDs and a PDA |
|  | 68  (*patient 2*) | F | 0 months  *Prenatal onset, AAD: 11 days.* | Type 1 | homozygous del. of SMN1 | 1 copy | Common atrium and a PDA |
|  | 69  (*patient 4*) | F | 4 months  *Prenatal onset, AAD: 122 days.* | Type 1 | homozygous del. of SMN1 | 1 copy | Large ASD (secundum type), a subaortal VSD and a dilated right ventricle.  *Dilatation of the RV is a secondary effect to the presence of the ASD, which increases the blood volume to the RV and in turn causes dilatation.* |
|  | 70 | F | *AAD: 11 months* | Type 1 | homozygous del. of SMN1 | 2 copies | Small VSD and a PDA |
| Araujo (**2009**)[49] | 71  (*case 1*) | F | Onset at 0 months  *Prenatal onset, no info on survival* | Type 1 | homozygous del. of SMN1 | n/a | ASD and asymmetric ventricular hypertrophy  *The ventricular hypertrophy is most likely acquired (secondary), not a primary cardiac abnormality.* |
| Lumaka (**2009**)[50] | 72 | M | Onset at 0 months, presentation at 5.5 months.  *AAD: 10 months* | Type 1 | homozygous del. of SMN1 | 2 copies | ASD (secundum type). |
| Rüdnik-Schöneborn (**2010**)[51] | 73  (*patient 1*) | M | 0 months.  *Onset from birth, AAD: 14 months* | Type 1 | homozygous del. of SMN1 | 1 copy | Large ASD, mild pulmonary and mild aortic stenosis |
|  | 74  (*patient 2*) | F | 0 months.  *Onset from birth, AAD: 7 months* | Type 1 | homozygous del. of SMN1 | 1 copy | AVSD |
| Parra (**2012**)[52] | 75^¥^ | n/a | n/a | Type 1 | homozygous del. of SMN1 | 1 copy | HLHS |
| Ekici (**2012**)[53] | 76 | M | 5.5 months  *AAD: 9 months.* | Type 1 | homozygous del. of SMN1 exons 7 + 8 | 2 copies | Dextrocardia and Tetralogy of Fallot |
| Khera (**2014**)[54] | 77 | M | 0 AAD: 108 days | Type 0 | Homozygous del. of SMN1 exons 7 + 8 | n/a/ | VSD |

Table S1: **^♦^**:The diagnosis as stated by the authors of the original publications is shown under ‘SMA type’. Case no.: sums up all included cases, *= patients within the study are siblings, ¥ = pregnancy was terminated due to presence of HLHS, ǂ = Two cases were presented in this article but one case was already published in 2006 and is therefore already in the list; Sex: m = male, f = female, n/a = information not available; Age (years): age at presentation as reported in the original publication. This overlaps with age at diagnosis in some publications; AAD = age at death; SMA type: WH = Werdnig-Hoffmann; SMN1 gene: n/a = info not available or no genetic testing performed, homozygous del. SMN1 ex 7 (+ 8) = SMA genetically confirmed based upon a homozygous deletion of SMN1 exon 7 (+ exon 8); SMN2 copy no: n/a = not available, χ = estimated copy number based on provided SMN:MPZ ratio by authors[38]; Cardiac pathology: all studies that were obtained after the search and selection process are shown here, including studies reporting ECG baseline abnormalities (nowadays regarded as artefacts due to the characteristic peripheral muscle tremor in SMA). ASD = atrial septal defect, VSD = ventricular septal defect, PDA = patent ductus arteriosus, HLHS = Hypoplastic Left Heart Syndrome, AVSD = atrioventricular septal defect. For reasons of completeness cases presenting with ECG (baseline) tremors are also shown under ‘cardiac pathology’, but we now know that these ECG tremors are caused by muscle tremors and do not reflect actual cardiac pathology.

**Table S2: overview of included studies on cardiac pathology in SMA type 2.**

| **Reference** | **Case no.** | **Patient characteristics** | | | **SMN1 gene status** | **SMN2 copy no.** | **Cardiac pathology** |
| --- | --- | --- | --- | --- | --- | --- | --- |
|  |  | **Sex (m/f)** | **Age (years)** | **SMA type** |  |  |  |
| Dawood (**1983**)[22] | 1–8 | n/a *M:F=1:1 for entire group of 10 patients* | n/a  *mean 2.6 years (age range 1.4–6 of entire group of 10 patients.* | SMA type 2 (‘intermediate form’) | n/a | n/a | ECG baseline tremors. |
| Coletta (**1989**)[25] | 9  (‘D.C.’) | F | 18 months | SMA type 2 (‘intermediate form’) | n/a | n/a | ECG baseline tremors in both limb and precordial (chest) leads, mild right ventricular dilatation. |
|  | 10  (‘V.C.M.’) | F | 5 months | SMA type 2 (‘intermediate form’) | n/a | n/a | ECG baseline tremors in limb leads, but not in precordial (chest) leads. |
|  | 11  (‘S.M.’) | F | 5.5 | SMA type 2 (‘intermediate form’) | n/a | n/a | ECG baseline tremors in both limb and precordial (chest) leads. |
|  | 12  (‘R.S.’) | F | 1.5 | SMA type 2 (‘intermediate form’) | n/a | n/a | ECG baseline tremors in both limb and precordial (chest) leads. |
|  | 13  (‘J.D.’) | F | 3 | SMA type 2 (‘intermediate form’) | n/a | n/a | ECG baseline tremors in both limb and precordial (chest) leads. |
|  | 14  (*‘C.L.’*) | M | 2.9 | SMA type 2 (‘intermediate form’) | n/a | n/a | ECG baseline tremors in both limb and precordial (chest) leads. |
|  | 15  (*‘D.S.’*) | M | 4 | SMA type 2 (‘intermediate form’) | n/a | n/a | ECG baseline tremors in both limb and precordial (chest) leads. |
|  | 16  (*‘B.M.’*) | F | 3.8 | SMA type 2 (‘intermediate form’) | n/a | n/a | ECG baseline tremors in both limb and precordial (chest) leads, mild septal dyskenesia. |
| Moosa (**1990**)[26] | 17–29 | n/a  *M:F=12:7 for entire group of 19 patients* | n/a  *Onset for entire group between 6 months and 2 years.* | SMA type 2  (‘intermediate form’) | n/a | n/a | ECG baseline tremors. |
| Carter (**1995**)[57] | 30–41 | n/a | n/a  *Age range of entire group of 32 patients: 17 ± 14 years* | SMA type 2 | n/a | n/a | ECG baseline tremors. Also abnormal Q-waves in 2 patients, increased R/S waves (lead: V3) in 7 patients, resting tachycardia in 2 patients, RBBB in 2 patients, non-specific ST-changes in 3 patients, atrial enlargement in 3 patients, ventricular enlargement in 4 patients. |
| Huang (**1996**)[34] | 42–63 | n/a  *M:F=1.7:1 for entire group of 27 patients* | n/a  *Age range of entire group of 27 patients: 39.8 ± 3.61 months* | SMA type 2 (‘intermediate form’) | n/a | n/a | ECG baseline tremors (16 continuous tremors, 6 intermittent ECG tremors) |

Table S2: Case no.: sums up all included cases; Sex: m = male, f = female, n/a = no information available; SMN1 gene: n/a = info not available or no genetic testing performed, homozygous del. SMN1 ex 7 (+ 8) = SMA genetically confirmed based upon a homozygous deletion of SMN1 exon 7 (+ exon 8); SMN2 copy no: n/a = info not available; Cardiac pathology: all studies that were obtained after the search and selection process are shown here, including studies reporting ECG baseline abnormalities (nowadays regarded as artefacts due to the characteristic peripheral muscle tremor in SMA). RBBB = right bundle branch block; IVS = interventricular septum; PW = posterior wall. For reasons of completeness cases presenting with ECG (baseline) tremors are also shown under ‘cardiac pathology’, but we now know that these ECG tremors are caused by muscle tremors and do not reflect actual cardiac pathology.

**Table S3: overview of included studies on cardiac pathology in SMA type 3.**

| **Reference** | **Patient characteristics** | | | | **SMN1 gene status** | **SMN2 copy no.** | **Cardiac pathology** |
| --- | --- | --- | --- | --- | --- | --- | --- |
|  | **Case no.** | **Sex (m/f)** | **Age** | **SMA type** |  |  |  |
| Gardner-Medwin (**1967**)[58] | 1 | F | 12 years  *Age at onset < 3; AAD:  12 years* | SMA type 3  (probably type 3a) | n/a | n/a | A cardiomyopathy and hypertrophic, dilated heart.  Histology: myocardial fibrosis and hypertrophic myocardial fibers.  ECG suggestive of severe myocardial damage. |
| Sterz (**1971**)[17] | 2  (*‘case 1’*) | M | 35 | SMA type 3 (KW) | n/a | n/a | Atrial fibrillation, AV-block with idioventricular rhythm. |
|  | 3  (*‘case 2’*) | M | 29 | SMA type 3 (KW) | n/a | n/a | Atrial fibrillation and signs of cardiomegaly. |
| Matsumoto (**1971**)[18] | 4  (*‘case 1’*) | n/a | n/a | SMA type 3 (KW) | n/a | n/a | Cardiomegaly, atrial standstill, AV-nodal ventricular rhythm, low QRS voltages |
| Emery (**1972**)[59] | 5–30 | n/a | n/a  *In total, 20 SMA patients were >2 years, 9 were > 6 years.* | SMA type 3 (WKW) | n/a | n/a | ECG baseline tremor in 26 patients. R-S interval abnormalities in 2 of these patients.  *The algebraic sum of the R and S waves (R-S) in V1 was used as a measure of cardiac involvement. Studies in the mid-1960s had indicated that this was a useful index for distinguishing certain forms of dystrophy, after which it was also used in SMA. Nowadays this measurement is not in use anymore.* |
| Sugimura (**1973**)[19] | 31 | F | 23 | SMA type 3 (KW) | n/a | n/a | Supraventricular premature beats, first degree AV block, paroxysmal atrial tachycardia and AV-nodal rhythm on ECG.  Heart biopsy: fibrosis of the right atrium. |
|  | 32 | M | 31 | SMA type 3 (KW) | n/a | n/a | Signs of cardiomegaly, atrial fibrillation, premature atrial beats, left axis deviation, coronary type T waves, and a slurred R on ECG. |
| Nuruki (**1974**)[60] | 33 | n/a | n/a | SMA type 3 (KW) | n/a | n/a | On light microscopy (biopsy specimen): deranged atrophic myocardial cells, proliferation of interstitial tissues.  Ultrastructural studies revealed focal degeneration of the myocardial cells containing Z-like substances, large dense bodies and densely packed actin-like myofilaments. |
| Tomlinson (**1974**)[61] | 34  (*case 7, ‘R.S.’)* | M | AAD: 17 years | SMA type 3b (KW) | n/a | n/a | Severe cardiomyopathy. ECG changes indicating lateral myocardial infarction. Patient died due to cardiac failure. Autopsy: severe loss of myocardial muscle fibers, especially in the left ventricle. Widespread patchy and extensive fibrosis. |
|  | 35  (*case 9, ‘J.S.’)* | M | AAD: 57 years | SMA type 3b (KW) | n/a | n/a | ECG: left axis deviation, myocardial ischemia. Myocardial infarction at the age of 54 years. Chronic congestive heart failure. Autopsy: markedly enlarged heart size, dilatation of both ventricles, extensive myocardial fibrosis. Fibrosis due to coronary artery disease according to the authors. |
| Tanaka (**1976**)[62] | 36  (*‘case 1’*) | M | 24  *Disease onset at 6 years* | SMA type 3 (KW) | n/a | n/a | Systolic cardiac murmur II/VI. ECG: Atrial flutter with complete AV-block. His bundle electrogram reveals atrial flutter with complete AV block due to an A-H block.  Undulatory pattern of mitral valve leaflets, tricuspid valve leaflets and interventricular septum. Right ventricular and left atrial dimensions increased on echocardiography. |
|  | 37  (*‘case 2’*) | M | 26  *Disease onset at 16 years* | SMA type 3 (KW) | n/a | n/a | Cardiac murmur II/VI. ECG: AV-junctional rhythm, QRS axis of 0 degrees. Deep Q-waves in lead I, aVL, and V5 and V6, An RS in V1. Small notches in the QRS comples of II, III, aVL and aVF.  Interstitial fibrosis of the right ventricle. |
| Lagarde (**1976**)[20] | 38 | M | n/a | SMA type 3 | n/a | n/a | ECG: sings of LV overload. Pectoral angina, palpitations and cardiomegaly. |
| Tanaka (**1977**)[63] | 39 | M | 20  *Disease onset at 17 years* | SMA type 3 (KW) | n/a | n/a | Electron microscopy: Degenerated myocytes found in between healthy myocardial fibers.  In these degenerated myocardial fibers: preferential loss of myosin filaments, a large number of fine filaments packed in the cytoplasm. Thick filaments completely lost or very few in these cells. Also leptomeric fibrils present and some abnormal mitochondria. |
| Russman (**1979**)[21] | 40–55 | n/a | n/a  *Age range of entire group of 19 patients:  3–29 years* | SMA type 3 (‘childhood chronic SMA’) | n/a | n/a | ECG baseline tremors in all 16 patients.  *Authors mention that the baseline tremors are to be interpreted as ‘muscle tremor artifacts’. These are present on ECG of SMA patients both in presence and absence of a clinically observed muscle tremor.* |
| Kimura (**1980**)[64] | 56 | F | 21  *Disease onset at 4 years* | SMA type 3 (KW) | n/a | n/a | Cardiac systolic ejection murmur grade II/VI. Cardiac enlargement on X-ray.  ECG: bradycardia, atrial standstill and AV-junctional rhythm.  Left ventricular diastolic dimensions slightly increased. |
| Ceroni (**1982**)[65] | 57 | M | 49  *Disease onset ‘as a youngster’* | SMA type 3 (KW) | n/a | n/a | Pansystolic regurgitant cardiac murmur grade IV/VI. Overall enlargement of the heart on X-ray. ECG: atrial flutter with complete AV-block, high idioventricular rhythm with isolated ectopic beats, extreme left axis deviation as if due to left anterior hemiblock, Q waves in V1, V2 and V3.  Echocardiography: pansystolic mitral valve prolapse with multiple echoes in systole and wide excursion of the leaflets in diastole. Similar findings for the tricuspid valve. The interventricular septum and the posterior wall of the ventricles were sluggish with significant impairment of ventricular function. |
| Dawood (**1983**)[22] | 58–66 | n/a  *M:F=1.75:1 for entire group of 11 patients* | n/a  *12.3 years, age range of entire group of 11 patients: 2.5–32 years.*  *Age at onset: mean 2.8 years (range: 1– 12 years)* | SMA type 3 (‘mild’; KW) | n/a | n/a | ECG baseline tremors |
| Hofstad (**1984**)[66] | 67–68 | n/a  *M:F=3.4:1 for entire group of 44 patients* | n/a  *54.3 years, age range for entire group: 19–77 years.* | SMA type 3 | n/a | n/a | Coronary heart disease |
|  | 69 |  |  |  |  |  | Hypertensive cardiopathy |
|  | 70 |  |  |  |  |  | Cardiac insufficiency |
|  | 71–76 |  |  |  |  |  | ECG abnormalities: **2x** signs of myocardial ischemia; **1x** left anterior hemiblock plus myocardial ischemia; **1x** left ventricular strain; **1x** right bundle branch block plus left anterior hemiblock; **1x** 2^nd^ degree AV block, Mobitz type I (Wenkebach) |
| Stegaru-Hellring (**1988**)[67] | 77–81 | n/a | n/a  *Mean age: 22.8 years for entire group of 5 patients, mean disease duration of 14.3 years* | SMA type 3 (KW) | n/a | n/a | All 5 patients had a prolonged QT-time interval on ECG. Further findings included 3 patients with mitral valve prolapse, 2 with a hypertrophic IVS, 1 with posterior wall hypertrophy, 1 with posterior wall atrophy and 1 with a diminished left ventricle end diastolic volume.  *Note: some patients had more than 1 structural abnormality.* |
| Coletta (**1989**)[25] | 82  (*‘R.V.’*) | F | 24 | SMA type 3 (KWD) | n/a | n/a | ECG baseline tremors in both limb and precordial leads. |
| Moosa (**1990**)[26] | 83–88 | n/a  *M:F=5:4 for entire group of 9 patients* | n/a  *Onset ranges between 6 months and 4 years for entire group.* | ‘Juveline type’ (Kugelberg-Welander) | n/a | n/a | ECG baseline tremors. |
| Bataille (**1991**)[68] | 89 | n/a | 10 | SMA type 3 (KW) | n/a | n/a | ECG: atrial rhythm disorder |
| Carter (**1995**)[57] | 90–103 | n/a | n/a  *Age range of entire group of 13 patients: 40 ± 20 years.* | SMA type 3 | n/a | n/a | ECG baseline tremors in all 14 patients. Additionally: abnormal Q-waves in 3 out of 14 patients, increased R/S waves (V3) in 11 out of 14 patients, resting tachycardia in 1 out of 14 patients, RBBB in 1 out of 14 patients, non-specific ST-changes in 2 out of 1 4 patients, signs of atrial enlargement in 1 out of 14 patients. |
| Mulleners (**1996**)[32] | 104  (‘*case 2’*) | F | 2.2 years | SMA type 3a | homozygous del. SMN1 ex 7 | n/a | ASD (ostium secundum type), L-TGA, functional single ventricle, PDA. |
| Huang (**1996**)[34] | 105–114 | n/a  *M:F=1.6:1 for entire group of 13 patients* | n/a  *Age range for entire group of 13 patients: 5.1 ± 3.6 years.* | SMA type 3 (‘mild’) | n/a | n/a | ECG baseline tremors (9 continuous tremors, 1 intermittent tremors) |
| Elkohen (**1996**)[69] | 115  (*‘observ. no. 8’)* | M | 14 | SMA type 3 (KW) | n/a | n/a | ECG abnormalities: AV-block (variable 1^st^ and 2^nd^ degree block), junctional escape rhythm, isorhythmic dissociation and sinus dysfunction. |
|  | 116  (*‘observ. personelle’)* | M | 20 | SMA type 3 (KW) | n/a | n/a | Dilating cardiopathy with fractional shortening: 15%.  ECG: ventricular extra systoles, signs of left ventricular overload. |
| Liu (**1999**)[70] | 117 | M | 37  *Onset in childhood* | SMA type 3 (KW) | n/a | n/a | ECG: Atrial standstill and prolonged junctional recovery time. |
| Yasuma (**2004**)[71] | 118 | M | 53  *Onset from early teens* | SMA type 3 (KW) (type 3b) | homozygous del. SMN1 exons 7 + 8 | n/a | Diffused and severe hypokinetic motion of the LV with abnormal LV ejection fraction (29,2%) and abnormal LV end diastolic diameter. Cardiomegaly present on X-ray. |
| Takahashi (**2006**)[72] | 119 | M | 51  *Age at onset: 30 years* | SMA type 3 (KW) | n/a | n/a | ECG: 2:1 AV block and a complete RBBB. Progression to complete AV-block without escape rhythm. His electrogram: his bundle-ventricular block.  Cardiomegaly on chest X-ray |
| Roos (**2009**)[73] | 120* | M | 43 | SMA type 3 (‘adult onset KWD’) | no SMN1 mutations | n/a | ECG: first degree AV block and left anterior hemi-block at age 33. Progression towards NSVTs with ventriculo-atrial dissociation. LV ejection fraction of 45% with an akinetic apical region of the LV and a pacemaker induced dyssynchrony. |
|  | 121* | F | n/a | SMA type 3 (‘adult onset KWD’) | n/a | n/a | ECG: first-degree AV block and paroxysmal episodes of atrial fibrillations as well as NSVTs. |
| Kuru (**2009**)[74] | 122^◊^ | M | *Onset at 14 years,  AAD: 67 years* | SMA type 3  (type 3b) | homozygous del. SMN1 exons 7 + 8 | n/a | Ventricular enlargement and diffuse hypokinesia.  Post mortem findings show mild myocardial fibrosis. |
| Vilela (**2012**)[75] | 123 | M | 61 years | SMA type 3 (KW) | n/a | n/a | ASD (ostium secundum type) |
| Haliloglu (**2015**)[76] | 124 | M | 15 years | SMA type 3 | Homozygous SMN1 deletion | n/a | Baseline tremors. |

Table S3: Case no.: sums up all included cases, *= patients within the study are siblings, ◊ = this patient is a brother of the male patient reported by Yasuma (2004); Sex: m = male, f = female, n/a = information not available; Age: AAD = age at death. SMA type: KW = Kugelberg-Welander, WKW = Wolfhart-Kugelberg-Welander. The SMA type as shown in the original publications is shown in the table. Nowadays we would re-classify some of the diagnoses (e.g. to SMA type 3b or SMA type 4); SMN1 gene: n/a = info not available or no genetic testing performed, homozygous del. SMN1 ex 7 (+ 8) = SMA genetically confirmed based upon a homozygous deletion of SMN1 exon 7 (+ exon 8); SMN2 copy no: n/a = info not available; Cardiac pathology: AV-block = atrioventricular conduction block, RBBB = right bundle branch block, ASD = atrial septal defect, L-TGA = left transposition of the great arteries, PDA = patent ductus arteriosus, IVS = interventricular septum, PW = posterior heart wall, E/A-ratio <1 indicates diastolic dysfunction, LV = left ventricle, NSVT = non sustained ventricular tachycardia. For reasons of completeness cases presenting with ECG (baseline) tremors only were also included in the table.

**Table S4: Overview of possibly relevant studies that were not available full text.**

| **Reference** | | |
| --- | --- | --- |
| **Authors** | **Title** | **Retrieved from** |
| Nixon (**1927**)[115] | Early infantile progressive muscular atrophy (Werdnig-Hoffmann) a clinical and pathologic study of two cases. | Original search |
| Sterne (**1964**)[116] | Cardiac manifestations during diseases of the nervous and muscular systems. | Original search |
| Brandt (**1950**)[117] | Werdnig-Hoffmann's infantile progressive muscular atrophy; clinical aspects, pathology, heredity, and relation to Oppenheim's amyotonia congenita and other morbid conditions with laxity of the joints or muscles in children. | References search* |
| Kohn (**1968**)[118] | Postmortem findings in a case of Wohlfart-Kugelberg-Welander disease. | References search* |
| Meunier-Carus (**1974**)[119] | Functional cardio-respiratory repercussions of the neuromuscular diseases of the thoraco-abdominal wall. | Original search |
| Okazaki (**1976**)[120] | Anestheic management of patients with Kugelberg-Welander disease associated with heart disease. | Original search |
| Ikuta (**1979**)[121] | Postmortem findings in a case of KW disease; presence of underdeveloped schwann cells and axons. | References search* |
| Popov'ian (**1984**)[122] | Myocardial lesions in several forms of progressive muscular atrophy. | Original search |
| Silva (**1987**)[123] | Cardiorespiratory responses to exercise in patients with spinal muscular atrophy and limb-girdle dystrophy. | Original search |
| Carboni (**1988**)[124] | Usefulness of ECH in the early diagnosis of infantile spinal muscular dystrophy. | Original search |
| Gupta (**1989**)[125] | Spinal muscular atrophy: some easy clues to diagnosis. | Original search |
| Malcic (**1991**)[126] | Cardiomyopathies in children with neuromuscular disorders. | Original search |
| Zupan (**1995**)[127] | Cardiovascular aberrations in patients with neuromuscular diseases. | Original search |
| Stöllberger (**2001**)[128] | Echocardiography in storage and neuromuscular disorders. | Original search |
| Matkowska-Kocjan (**2008**)[129] | Vaccinations in children’s palliative care – Our proposal for the algorithm. | Original search |

Table S4: overview of articles (*n* = 12) possibly relevant to our systematic search of which no full text or detailed abstract was available. Three articles were retrieved either using the online Thomson Reuters’ “Web of Science” tool for a cited references search (‘related articles search’), or through checking reference lists of all included full text articles from our initial search.

**Table S5: Overview of SMA mouse models with cardiac pathology**

| **Cardiac pathology in SMA mouse models** | | | | | | |
| --- | --- | --- | --- | --- | --- | --- |
| **References** | **Mouse model^ǂ^** | **Macroscopic cardiac abnormalities** | **Microscopic / Histologic cardiac abnormalities** | **Abnormalities of cardiac rhythm** | **Functional cardiac abnormalities** | **Remarks** |
| Bevan A.K. (**2010**)[77] | SMNΔ7  (*Smn* ^–^/^–^, SMN2^+/+^, SMNΔ7^+/+^; **s*evere*** *model*) | Decreased heart size, indicated by decreased LV mass, thinner cardiac walls. Eccentric cardiac hypertrophy, development of dilating cardiomyopathy and fatal congestive heart failure. | Mitochondria in cardiomyocytes swollen and degenerative. Marked disorganization and degeneration of myofibers. Swollen myofibers also present. Myelin bodies throughout tissue present, indicating organelle and/or membrane turnover implying significant cardiac dysfunction. | Severe sinus bradycardia | Lower stroke volume, lower cardiac output, lower fractional shortening, increased LV-Tei index indicating overall decreased function. | Decreased heart size/mass and subsequent functional  abnormalities are in part caused by smaller body size and do not all represent SMA-specific cardiac pathology. |
| Heier C.R. (**2010**)[78] | SMNΔ7  (JAX line 5025; ***severe*** *model*) | Smaller and flaccid SMA mouse hearts, lacking defined shape with gross attenuation of walls.  Sings of a dilating cardiomyopathy at later stages of the disease. | Abnormalities of autonomic cardiac innervation: nerve staining indicates reduced neuronal branching, thinner and less prominent sympathetic autonomic cardiac neurons.  *This could cause an autonomic imbalance with decreased sympathetic tone and increased parasympathetic (vagal) tone, contributing to cardiac arrhythmias.* | Severe bradyarrhythmias, already present from early postnatal stages. Reduced ventricular depolarization efficiency and progressive heart block. Progression from severe bradycardia to cardiac standstill at end stages of life. | Significant reductions in pumping efficiency and blood flow from the RV to the lungs. | Effects on heart rate or ECG waveforms as reported are not caused by transgene insertion or *Smn* heterozygosity. |
| Shababi M. (**2010**)[79] | SMNΔ7  (*Smn* ^–/–^, SMN2^+/+^, SMNΔ7^+/+^; **s*evere*** *model*) | Abnormal embryonic cardiac remodelling of IVS, LV and arterial walls causing reduced LV wall width, LV lumen enlargement, severe postnatal reduction of IVS width. | Myocardial interstitial fibrosis causing abnormal myocardial stiffness and decreased cardiac function. Rapid postnatal progression of fibrosis was noted, increased angiotensin-II-mediated oxidative stress was identified as the underlying cause.  Partial flattening and thinner arterial walls, not explained by smaller body/heart size. | Significant slower heart rate in SMA mice. | – | Reduced size of the heart due to small body mass (rather than due to irregular loss of growth of the heart). |
| Hua Y.  (**2011**)[10] | Smn^–/–^ SMN2^+/0^  (JAX stock number TJL-005058; ***severe*** *model)* | Decreased heart weight, decreased width of IVS and LV. | – | – | – | – |
| Gogliotti R.G. (**2012**)[80] | *Tg*(SMN2)89Ahmb/J, *Smn1^tm1Cdid/ tm1Cdid^*  or *(SMN2)89Ahmb^+/–^, Smn^2B-Neo/2B-Neo^*  *(‘****severe*** *inducible model’)* | – | Abnormalities of autonomic cardiac innervation: nerve staining indicates significant reduced neuronal branching. | Significant severe progressive bradycardia at later time points. Signs of terminal heart block, delays in electrical conductance system. | – | Autonomic innervation defects exist in the absence of significant paralysis and/or motor unit pathology, suggesting that these are primary effects and additively contribute to the poor health of SMA mice. |
| Biondi O. (**2012**)[81] | *Smn^Δ7/ Δ7^*; SMN2^+^*^/^*^−^  *(*Academia Sinica, Taipei, Taiwan;  ***SMA type 2*** *model)* | Atrophic cardiac ventricles, thin IVS and disorganized isolated round and atrophic cardiomyocytes. | Abnormal expression of markers of postnatal cardiac development indicating loss of contractile components.  High proportion of myocardial fibrosis was observed. | Progressive bradycardia and cardiac conduction defects indicated by prolonged SA to AV conduction time and increased QRS duration.  Non-pathological sympathetic activity counteracts alteration of cardiac function, such as arrhythmia and reduced heart rate | – | Physical exercise partially improved both functional and structural parameters.  Bradycardia proportional to the drop in body weight at later time points. |
| Osborne M. (**2012**)[82] | SMN*1^C/C^*  (JAX stock number 8604; ***mild*** *phenotype*) | – | – | Significant slower heart rate. | – |  |
| Shababi (**2012**)[83] | SMNΔ7  (*mSmn–/–, hSMN2+/+, SMNΔ7+/+;* **s*evere*** *model*) | IVS remodelling resulting in dilated ventricles. | Significant reduction in cardiomyocyte size in SMA mice. Cardiac fibrosis present at early time-points, caused by high levels of reactive oxygen species. Loss of smooth muscle cells in walls of arterioles, leading to reduced thickness of walls of arterioles. Reduced number of myocardial capillaries and consequently a reduced number of capillaries expressing von-Willebrand Factor. | – | *Cardiac function assessed in SMA mice after treatment with scAAV-SMN at a time point where untreated mice had already deceased.*  Insufficient cardiac function was indicated by a decreased heart rate, stroke volume, cardiac output, and ejection fraction. Findings also included a reduced LV myocardial mass. | – |
| Schreml J. (**2013**)[84] | SMN*^–/–^*;SMN2*^tg/0^*  *(****severe*** *model)* | Thinner IVS. | – | – | – | Cardiac abnormalities already present before onset of motor symptoms. |
| Cobb M.S. (**2013**)[85] | SMN*^RT^*  (SMN2+/+; SMN^RT^; Smn–/–; ***intermediate*** *SMA model*) | Slightly reduced IVS thickness, but not statistically significant. | Significant modest increase in myocardial fibrosis. | – | – |  |
| Keil J.M. (**2014**)[86] | 5058-Hemi SMA mice  (SMN2^tg/0^; *Smn*^tm1Hung/tm1Hung^; ***severe*** *model*) | – | – | Bradyarrhythmia (lower heart rate, longer PR interval), Elongated QRS interval indicative of delayed ventricular depolarization times, suggesting a bundle branch block.  *Authors conclude that the bradyarrhythmia and heart block are suggestive of sympathetic innervation deficits in the 5058-Hemi hybrid SMA mice.* | – | – |
| Tsai L-K (**2014**)[87] | SMN*^–/–^*;SMN2^+^*^/–^*  *(****severe*** *model)* | Lower heart weight, reduced thickness of LV, reduced width of IVS. | – | – | – | – |
| Heier (**2015**)[88] | SMNΔ7  (Jackson Laboratories, strain 605025; ***severe*** *model*) | - | - | 1. Significant lower heart rate compared to unaffected littermates.  2. Signs of heart block (elongated PR-interval) in affected mice.  3. Impaired cardiac conduction (significantly slower QRS intervals) |  | Signs of significant alterations to the autonomic nerve control of the heart in SMA mice were also found. |
| Bogdanik (**2015**)[89] | Burgheron mice  (Jax Stock 14561, FVB.Cg-Tg(SMN2) 89Ahmb Smn1^tm1Msd^  *S*mn1^tm5(Smn1/SMN2)Mrph^/J; ***intermediate*** *model, to represent SMA types II/III*) | Left ventricular hypertrophy, smaller overall heart size with both ventricular and atrial dilatation. |  | Significant slower heart rate. | Lower ejection fraction | A distinction is made between the ‘short-lived’ Burgheron mutants, with a severe cardiomyopathy, and a longer surviving Burgheron mutant with less severe cardiac pathology. |

Table S5: Overview of articles (*n* = 14) on cardiac pathology in SMA model mice. ǂ: for specific details on the type of mouse model used (as indicated in the table) see the cited references. LV: left ventricle; RV: right ventricle; ANS: autonomous nervous system; IVS: inter-ventricular septum; ASO: antisense oligonucleotide; SA: sinoatrial; AV: atrioventricular; scAAV9: self-complementary adeno-associated virus; WT: wild type (i.e. unaffected).
